# The Significance of Somatic Variants Involved in the Development of Pancreatic Cancer in Patients with HBOC-Associated Germline Variants

**DOI:** 10.3390/genes17091146

**Published:** 2026-09-18

**Authors:** Koki Uchiyama, Hinano Nishikubo, Tomoya Sano, Yukina Furukawa, Dongheng Ma, Daiki Imanishi, Takemi Ishidate, Hongdong Gao, Canfeng Fan, Masakazu Yashiro

**Affiliations:** 1 Molecular Oncology and Therapeutics, Osaka Metropolitan University Graduate School of Medicine, 1-4-3 Asahimachi, Abeno-ku, Osaka 545-8585, Japan; si26985v@st.omu.ac.jp (K.U.); sn23089k@st.omu.ac.jp (H.N.); sb24524y@st.omu.ac.jp (T.S.); se26752s@st.omu.ac.jp (Y.F.); sg24231c@st.omu.ac.jp (D.M.); sy23003h@st.omu.ac.jp (D.I.); st26829x@st.omu.ac.jp (T.I.); se26546i@st.omu.ac.jp (H.G.); y23703o@omu.ac.jp (C.F.); 2 Cancer Center for Translational Research, Osaka Metropolitan University Graduate School of Medicine, 1-4-3 Asahimachi, Abeno-ku, Osaka 545-8585, Japan; 3 Department of Gastroenterological Surgery, Osaka Metropolitan University Graduate School of Medicine, 1-4-3 Asahimachi, Abeno-ku, Osaka 545-8585, Japan

**Keywords:** C-CAT, hereditary cancer, HBOC, pancreatic cancer, somatic variant, *KRAS*, TP53, germline variant, *BRCA1*, *BRCA2*, *ATM*

## Abstract

Background: Hereditary cancer predisposition syndromes show a high risk of cancer development with germline pathogenic variants. Organ-specific surveillance has been recommended according to the causative gene. However, even among carriers of the same germline variants, developing cancer types are not always uniform. Tumor–normal paired comprehensive genomic profiling (CGP) enables the simultaneous assessment of germline and somatic variants in same patient. Correlations between germline variants and cancer types have been reported; however, the significance of combinations of a germline variant and somatic variants remain unclear in the development of cancer types. Methods: In this study, we analyzed the correlation between pancreatic cancer and somatic variant patterns in patients with hereditary cancer predisposition syndrome, especially in patients with germline variants on *BRCA1*, *BRCA2*, or *ATM*. Results: Here, we show the characteristic pancreatic cancer patterns according to co-existing somatic variants in patients with germline pathogenic variants. The tumor–normal paired genetic data from 18,732 patients who underwent tumor–normal paired CGP were analyzed, including 853 germline-positive patients registered in a Japanese database. The combination of the somatic *TP53* variant and *KRAS* variant was closely correlated with the development of pancreatic cancer in patients with germline *BRCA1*, *BRCA2*, or *ATM*. In particular, the somatic *KRAS* variant was significantly associated with pancreatic cancer. Conclusions: These findings suggest that pancreatic cancer of hereditary cancer syndromes might be associated with co-existing somatic *KRAS* variants within the pancreas.

## 1. Introduction

It has been reported that pathogenic germline variants were identified in 4.3% of patients [1]. A pan-cancer analysis of 10,389 patients across 33 cancer types reported pathogenic or likely pathogenic germline variants in 8% of patients [2]. Hereditary breast and ovarian cancer syndrome (HBOC), a major hereditary cancer syndrome known to confer an increased risk of breast and ovarian cancer, is primarily associated with pathogenic germline variants in *BRCA1* or *BRCA2* [3,4]. Beyond the established causative genes *BRCA1* and *BRCA2*, additional candidate susceptibility genes have also been identified. These include *ATM* and *CHEK2*, which are involved in the DNA damage checkpoint, as well as *PALB2*, *BRIP1*, and *RAD51C*, which are involved in DNA double-strand break repair. Variants in these genes have been reported to be associated with an increased risk of breast cancer [5,6,7,8,9,10].

Carriers of pathogenic germline variants in *BRCA1* or *BRCA2* are at increased risk not only of breast and ovarian cancer but also of other malignancies, including pancreatic cancer and prostate cancer [11,12,13]. In particular, a large-scale family-based analysis reported that *BRCA1* pathogenic variants were associated with an increased risk of pancreatic cancer, whereas *BRCA2* pathogenic variants were associated with increased risks of pancreatic cancer and prostate cancer [13]. However, the reasons responsible for the development of different cancer types in HBOC patients remain unclear. Although environmental factors have been reported to influence cancer risk in HBOC patients, it remains unclear the relationship between somatic gene alterations and cancer development site in patients with hereditary cancer predisposition syndrome [14,15].

In this study, we aimed to clarify the significance of somatic variants involved in the development of pancreatic cancer of patients with HBOC-associated germline variant, especially *BRCA1*, *BRCA2*, or *ATM*.

## 2. Materials and Methods

### 2.1. Study Cohort and CGP Assays

A total of 120,364 patients were registered in the Center for Cancer Genomics and Advanced Therapeutics (C-CAT) database between June 2019 and March 2026. Among these, we selected cases analyzed using one of two tumor–normal paired comprehensive genomic profiling (CGP) tests: the GenMineTOP^®^ Cancer Genome Profiling System (GMT; Konica Minolta REALM, Inc., Tokyo, Japan) and the OncoGuide™ NCC Oncopanel System (NCC; Sysmex Corporation, Kobe, Japan). For GMT, DNA and RNA extracted from tumor tissue and DNA extracted from peripheral blood were analyzed. For NCC, DNA extracted from tumor tissue and peripheral blood was analyzed. The GMT DNA panel covered 737 genes [16], whereas the NCC panel covered 114 genes [17]. A total of 18,732 patients who underwent tumor and normal paired testing were registered. The analysis included 8109 GMT patients and 10,623 NCC patients. Cancer types were classified according to the organ site registered in the C-CAT database. This study was approved by the C-CAT review committee (C-CAT management number: CDU2022-044E04) and the Medical Ethics Committee of Osaka Metropolitan University (approval no. 2022-111) on 15 September 2022.

### 2.2. Variant Selection and Classification

CGP assays using next-generation sequencing (NGS) can detect nucleotide substitutions, insertions/deletions, copy-number alterations, and gene fusions. In the present study, the analysis was restricted to small-scale variants, including nucleotide substitutions and small insertions/deletions.

Variants were annotated by C-CAT as pathogenic, likely pathogenic, oncogenic, likely oncogenic, or variants of uncertain significance (VUS). Pathogenic, likely pathogenic, and VUS classifications were based on the sequence variant interpretation guidelines established by the American College of Medical Genetics and Genomics and the Association for Molecular Pathology (ACMG/AMP), whereas oncogenic and likely oncogenic classifications were based on C-CAT-specific criteria for clinical significance.

For germline analyses, pathogenic and likely pathogenic variants were included, whereas VUS were excluded. Patients harboring at least one pathogenic or likely pathogenic germline variant were defined as germline-positive patients. For somatic analyses, variants classified as pathogenic, likely pathogenic, oncogenic, or likely oncogenic were included.

### 2.3. Definition and Comparison of Gene-Level Germline–Somatic Variant Patterns

We focused on a *BRCA*-associated germline such as *BRCA1*, *BRCA2*, *ATM*, *PALB2*, *CHEK2, BRIP1, and RAD51C* in this study. Among these *BRCA*-associated genes, germline pathogenic valiant of *BRCA1*, *BRCA2*, and *ATM* were identified in more than 50 patients, but *PALB2 CHEK2*, *BRIP1* and *RAD51C* were less than 50 patients. Since over 50 patients were necessary for statistical analysis, *PALB2 CHEK2*, *BRIP1* and *RAD51C* were excluded in this study.

Among germline-positive patients, exact germline–somatic variant patterns were defined, and all observed exact patterns were included in the analysis. First, within carriers of germline variants in each gene, the frequency of pancreatic cancer was compared between patients with each indicated exact pattern and those with any other exact pattern. Odds ratios were calculated by comparing each indicated exact pattern with all other exact patterns. Second, pancreatic cancer frequencies were directly compared between nested exact pattern pairs that differed only by the addition of a single somatic variant gene. For these comparisons, odds ratios were calculated by comparing the pattern containing the additional somatic variant gene with the corresponding pattern without that gene. For patients with multiple primary cancers, only the organ site registered as the primary cancer was included in the cancer-type analysis.

### 2.4. Statistical Analysis

Age was compared using the two-sided Wilcoxon rank-sum test, whereas categorical variables were compared using Pearson’s chi-squared test or Fisher’s exact test, as appropriate. For descriptive cohort comparisons, a two-sided *p* < 0.05 was considered statistically significant. Odds ratios (ORs) and 95% confidence intervals (CIs) were calculated using the Wald method on the log odds-ratio scale after applying the Haldane–Anscombe correction, with 0.5 added to each cell. To account for multiple testing in the exact germline–somatic variant pattern analyses, the 244 exact-pattern comparisons and the 213 nested-pattern comparisons were treated as two separate testing families and were independently adjusted using the Benjamini–Hochberg (BH) method. Adjusted *p* values are presented as *q* values, with *q* < 0.05 considered statistically significant after multiple-testing correction. All statistical analyses and generation of figures and tables were performed using R version 4.6.0 (R Foundation for Statistical Computing, Vienna, Austria).

### 2.5. Sensitivity Analyses

To assess the effect of panel coverage, a common-114-gene (C114) analysis was performed, and when information was available from both panels, a panel-adjusted analysis using the exact Cochran–Mantel–Haenszel test was performed (Supplement Tables S1A,B). Detailed pancreatic tumor classifications were summarized in Supplement Table S2A, and sensitivity analyses restricted to cases registered as “Pancreatic Adenocarcinoma” were performed for the exact-pattern and nested-pattern analyses (Supplement Tables S2B,C).

## 3. Results

### 3.1. Germline-Positive Patients

**Figure 1:**
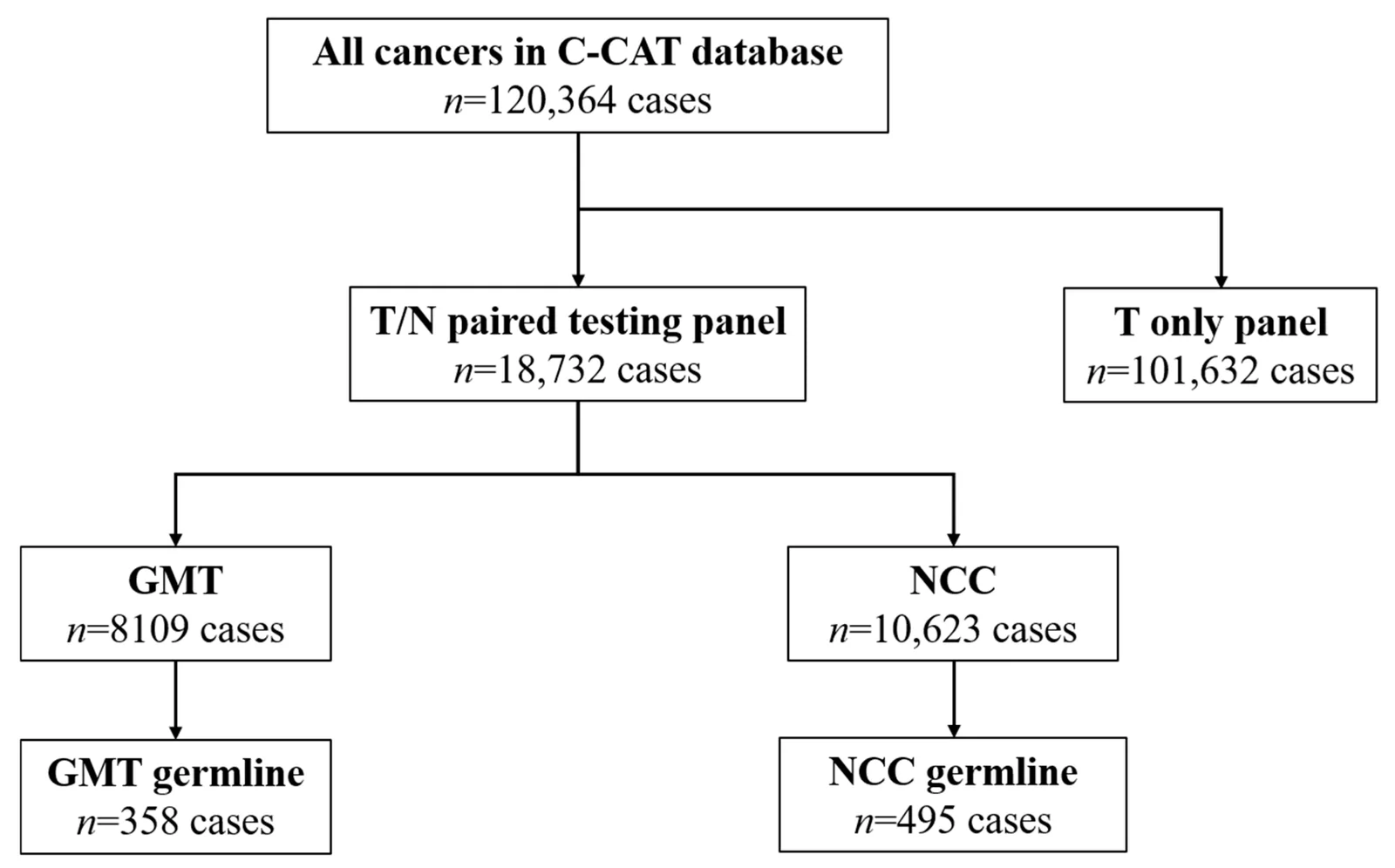
Study cohort and identification of germline-positive patients. Flow diagram of the study cohort derived from 120,364 patients registered in C-CAT database. T only testing was performed in 101,632 patients, whereas 18,732 underwent T/N paired testing and were included in the present analysis. Among these, 8109 patients underwent testing with GMT, of whom 358 (4.41%) were germline-positive, and 10,623 underwent testing with NCC, of whom 495 (4.66%) were germline-positive. In total, 853 patients were germline-positive. C-CAT, Center for Cancer Genomics and Advanced Therapeutics; T/N, tumor–normal; T only, tumor–only; GMT, GenMineTOP Cancer Genome Profiling System; NCC, OncoGuide NCC Oncopanel System.

Of the 120,364 cases registered in the C-CAT database, 18,732 cases underwent tumor–normal (T/N) paired testing including 8109 cases of GMT and 10,623 cases of NCC. Among these patients, 358 (4.41%) cases of GMT and 495 (4.66%) cases of NCC were germline-positive, yielding a total of 853 (4.55%) germline-positive patients among the 18,732 patients (Figure 1). The clinical characteristics of the 18,732 patients are summarized in Table 1. Germline-positive patients were significantly younger than germline-negative patients (mean age, 56.5 vs. 59.9 years; *p* < 0.001) and were more frequently female (53.6% vs. 49.4%; *p* = 0.018). Pancreatic cancer was significantly more frequent among germline-positive patients than among germline-negative patients (23.4% vs. 19.6%; *p* = 0.006). Significant differences were also observed in bowel cancer, breast cancer, lung, ovary/fallopian tube cancer, and prostate cancer. A family history of cancer was significantly associated with germline-positive patients (80.8% vs. 69.5%; *p* < 0.001).

### 3.2. Gene Distribution of Germline and Somatic Variants

**Figure 2:**
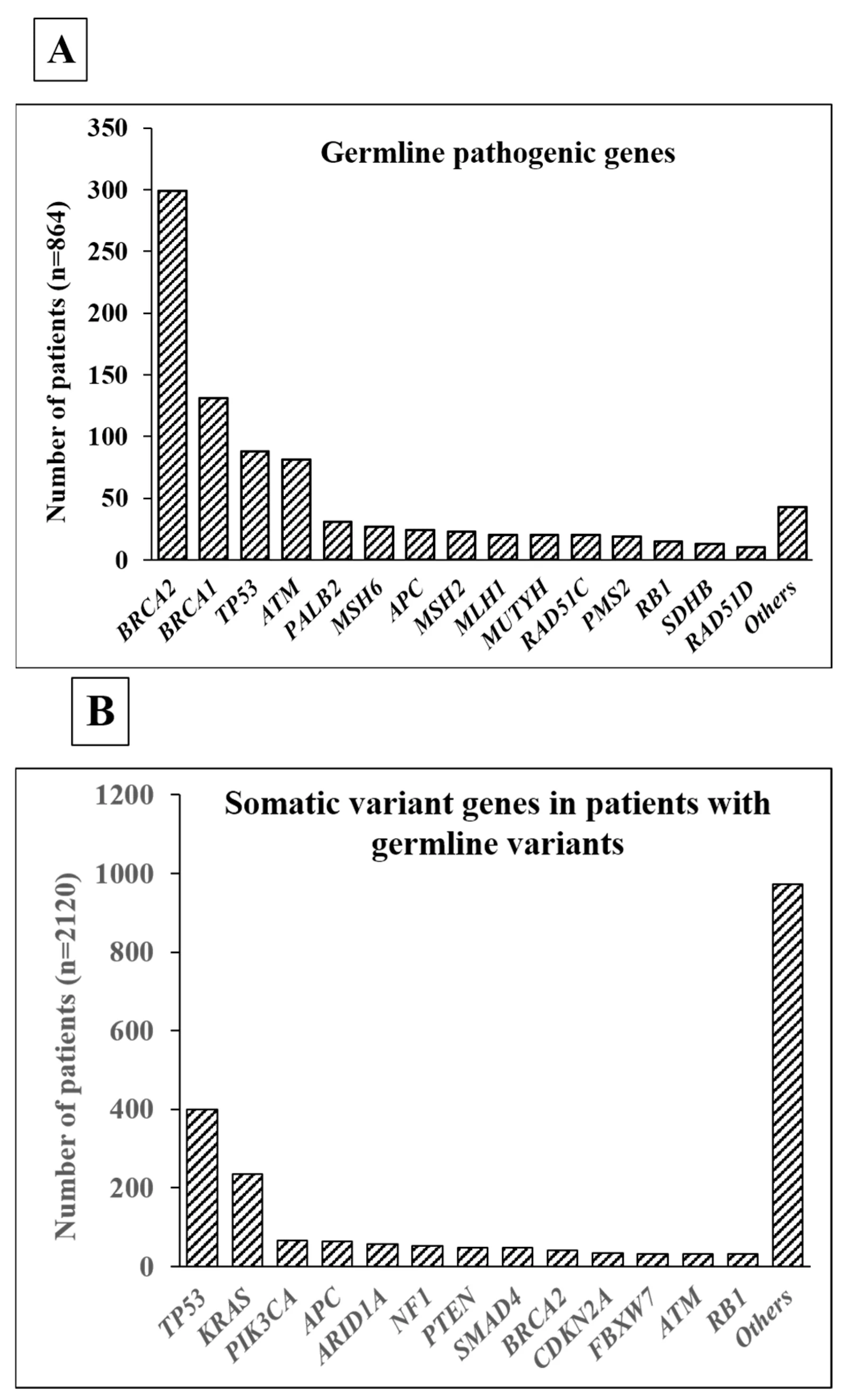
Gene-level distributions of germline and somatic variants. (**A**) Gene-level distribution of germline variants among the 853 germline-positive patients. When variants were detected in multiple genes in the same patient, each gene was counted separately, whereas multiple variants detected in the same gene in the same patient were counted only once. This resulted in a total of 864 patient–gene counts: *BRCA2*, 299; *BRCA1*, 131; *TP53*, 88; *ATM*, 81; *PALB2*, 31; *MSH6*, 27; *APC*, 24; *MSH2*, 23; *MLH1*, 20; *MUTYH*, 20; *RAD51C*, 20; *PMS2*, 19; *RB1*, 15; *SDHB*, 13; *RAD51D*, 10; and other genes, 43. (**B**) Gene-level distribution of somatic variants among the same 853 germline-positive patients. Somatic variants were counted using the same gene-level approach as in (**A**), resulting in a total of 2120 patient–gene counts: *TP53*, 400; *KRAS*, 235; *PIK3CA*, 66; *APC*, 65; *ARID1A*, 58; *NF1*, 52; *PTEN*, 49; *SMAD4*, 48; *BRCA2*, 42; *CDKN2A*, 35; *FBXW7*, 33; *ATM*, 32; *RB1*, 32; and other genes, 973.

Figure 2A shows the gene-level distribution of germline variants among the 853 germline-positive patients. The most frequent germline variant pattern was one variant per patient (839 cases), followed by two variants at different genes per patient (13 cases), and four variants within different genes (1 case), resulting in a total of 853 gene-level counts. Table 1 counts the number of individual germline variants, whereas Figure 2A uses patient–gene counts.

The most frequent germline variant gene was *BRCA2* (*n* = 299), followed by *BRCA1* (*n* = 131), *TP53* (*n* = 88), *ATM* (*n* = 81), *PALB2* (*n* = 31), *MSH6* (*n* = 27), *APC* (*n* = 24), *MSH2* (*n* = 23), *MLH1* (*n* = 20), *MUTYH* (*n* = 20), *RAD51C* (*n* = 20), *PMS2* (*n* = 19), *RB1* (*n* = 15), *SDHB* (*n* = 13), and *RAD51D* (*n* = 10). The remaining genes accounted for a total of 43 counts.

Figure 2B shows the somatic gene variants among the 853 patients with germline variant(s). Using the gene-level counting approach, each gene was counted once per patient, resulting in a total of 2120 gene-level counts. *TP53* had the highest count (*n* = 400), followed by *KRAS* (*n* = 235), *PIK3CA* (*n* = 66), *APC* (*n* = 65), *ARID1A* (*n* = 58), *NF1* (*n* = 52), *PTEN* (*n* = 49), *SMAD4* (*n* = 48), *BRCA2* (*n* = 42), *CDKN2A* (*n* = 35), *FBXW7* (*n* = 33), *ATM* (*n* = 32), and *RB1* (*n* = 32). The remaining genes accounted for a total of 973 counts.

### 3.3. Correlation Between Cancer Types and Germline–Somatic Variant Patterns

Among the 853 germline-positive patients, analyses were conducted separately for carriers of germline variants in each gene, as shown in Table 2.

#### 3.3.1. Carriers of Germline *BRCA1* Variants

Among carriers of germline *BRCA1* variants, pancreatic cancer was significantly less frequent in the pattern with a somatic *TP53* variant only, occurring in 0.0% (0/53) versus 16.7% (13/78) of patients (OR, 0.05; 95% CI, 0.00–0.78; *p* < 0.001, *q* = 0.034). In the pattern with somatic *KRAS* and *TP53* variants, pancreatic cancer was significantly more frequent, occurring in 83.3% (5/6) versus 6.4% (8/125) of patients (OR, 50.69; 95% CI, 7.32–350.95; *p* < 0.001, *q* = 0.003). Therefore, the pattern with somatic *KRAS* and *TP53* variants showed contrasting associations with pancreatic cancer.

#### 3.3.2. Carriers of Germline *BRCA2* Variants

In the pattern with a somatic *TP53* variant only, pancreatic cancer was significantly less frequent, occurring in 6.5% (2/31) versus 36.9% (99/268) of patients (OR, 0.14; 95% CI, 0.04–0.54; *p* < 0.001, *q* = 0.028). By contrast, in the pattern with somatic *KRAS* and *TP53* variants, pancreatic cancer was significantly more frequent, occurring in 100.0% (23/23) versus 28.3% (78/276) of patients (OR, 118.85; 95% CI, 7.13–1980.67; *p* < 0.001, *q* < 0.001).

#### 3.3.3. Carriers of Germline *ATM* Variants

Among carriers of germline *ATM* variants, pancreatic cancer was significantly more frequent in patterns containing a somatic *KRAS* variant. In *KRAS* variant cases, pancreatic cancer occurred in 90.9% (10/11) versus 34.3% (24/70) of patients with germline *ATM* variants (OR, 13.29; 95% CI, 2.24–78.74; *p* < 0.001, *q* = 0.028). In the pattern with somatic *ATM* and *KRAS* variants, pancreatic cancer occurred in 100.0% (8/8) versus 35.6% (26/73) of patients (OR, 30.47; 95% CI, 1.69–549.13; *p* < 0.001, *q* = 0.028).

### 3.4. Direct Comparison of Patterns Differing by a Single Additional Somatic Variant Gene

Next, among carriers of germline variants in the same gene, cancer-type distributions were directly compared between nested pattern pairs that differed by the addition of a single somatic variant gene. Hereafter, patient numbers are presented in the order of the pattern with the additional somatic variant gene and the corresponding pattern without gene (Table 3).

#### 3.4.1. Carriers of Germline *ATM* Variants

Among carriers of germline *ATM* variants, compared with the pattern without somatic variants, pancreatic cancer was significantly more frequent in the pattern with a somatic *KRAS* variant, occurring in 90.9% (10/11) versus 0.0% (0/10) of patients (OR, 147.00; 95% CI, 5.35–4037.73; *p* < 0.001, *q* = 0.002).

#### 3.4.2. Carriers of Germline *BRCA1* Variants

Among carriers of germline *BRCA1* variants, when a somatic *KRAS* variant was added to the pattern with a somatic *TP53* variant only, pancreatic cancer was significantly more frequent, occurring in 83.3% (5/6) versus 0.0% (0/53) of patients (OR, 392.33; 95% CI, 14.20–10,839.25; *p* < 0.001, *q* < 0.001).

#### 3.4.3. Carriers of Germline *BRCA2* Variants

Among carriers of germline *BRCA2* variants, when a somatic *TP53* variant was added to the pattern with a somatic *KRAS* variant only, pancreatic cancer was significantly more frequent, occurring in 100.0% (23/23) versus 55.6% (10/18) of patients (OR, 38.05; 95% CI, 2.00–722.26; *p* < 0.001, *q* = 0.024). In addition, when a somatic *KRAS* variant was added to the pattern with a somatic *TP53* variant only, pancreatic cancer was significantly more frequent, occurring in 100.0% (23/23) versus 6.5% (2/31) of patients (OR, 554.60; 95% CI, 25.38–12,120.45; *p* < 0.001, *q* < 0.001).

### 3.5. Supplementary Sensitivity Analyses

In the panel-coverage sensitivity analyses, the major associations observed in the primary analyses were generally maintained, although not all findings remained significant after multiple-testing correction (Supplement Tables S1A,B). Of the 3702 patients registered as having pancreatic cancer, 3024 (81.7%) were registered as “Pancreatic Adenocarcinoma,” including 165 of 200 (82.5%) germline-positive patients with pancreatic cancer (Supplement Table S2A). In the analyses restricted to this classification, the major findings were generally consistent with the primary analyses, although some associations did not remain significant after multiple-testing correction (Supplement Tables S2B,C).

## 4. Discussion

In this study, of the 120,364 cases registered in the C-CAT database, a total of 853 cases (4.55%) were germline-positive and considered to be hereditary cancer predisposition syndrome. The 853 germline-positive patients were younger and more frequently had a family history of cancer than the 17,879 germline-negative patients, consistent with previous reports [18,19]. In addition, pancreatic, breast, ovary/fallopian tube, and prostate cancers were significantly more frequent in the germline-positive group than in the germline-negative group. Although *BRCA1*, *BRCA2*, *TP53*, and *ATM* were among the most frequent germline variant genes, cancer types varied among carriers, suggesting that germline predisposition alone may not fully explain the organ site of cancer development. Then in this study, we analyzed the correlation between pancreatic cancer and somatic variant patterns in patients with hereditary cancer predisposition syndrome, with a particular focus on HBOC-related cancer predisposition in patients with germline variants in *BRCA1*, *BRCA2*, or *ATM*.

The present study demonstrated that the cancer-type of patients with hereditary cancer syndromes was associated with their co-existing somatic variants within the patients. Cancer-type differed according to the co-occurring somatic variant patterns in patients with the same germline variant. In HBOC patients with *BRCA1* germline variants, pancreas cancer was frequent in the pattern with co-occurring somatic *KRAS* and *TP53* variants. In HBOC patients with *BRCA2* germline variants, the somatic *KRAS*-only pattern was not significantly associated with pancreatic cancer after multiple-testing correction. Arafa et al. analyzed the somatic genomic landscape of tumors harboring germline variants, and reported that somatic *KRAS* mutations were significantly enriched in pancreatic cancer; in the germline *BRCA2* group [15]. These findings are consistent with our findings that specific patterns containing somatic *KRAS* mutations were associated with pancreatic cancer. The combination of variants, including the germline variant, may be associated with pancreatic cancer. Furthermore, when a somatic *TP53* variant was added to the somatic *KRAS* variant, pancreatic cancer was significantly more frequent. In patients with germline *ATM* variants, pancreatic cancer was frequent in patients with a *KRAS* variant. Also, when a somatic *KRAS* variant was accompanied by *ATM* variant, pancreatic cancer was frequent. These findings suggest that the site of developing tumor in HBOC patients might partially depend on the accompanying somatic alterations.

Taken together, the association between exact patterns and pancreatic cancer was not specific to combinations of germline *BRCA1*/2 variants with somatic *TP53* and *KRAS* variants but was also observed among carriers of germline *ATM* variants. These findings suggest that, to understand cancer-type distribution in hereditary cancer syndromes, it is important to evaluate the overall combination of germline and somatic variants within individual patients, rather than evaluating causative germline variants or individual somatic variants in isolation.

Germline *ATM* variants are known as genetic predispositions to pancreatic cancer, with carriers reported to have a 6.5-fold relative risk of pancreatic cancer compared with noncarriers [20]. However, that study estimated pancreatic cancer risk among carriers of germline *ATM* variants as a whole and did not address which molecular backgrounds among carriers are associated with the development of pancreatic cancer. In the present study, although all patients carried a germline *ATM* variant, pancreatic cancer was not observed in the pattern without somatic variants, whereas pancreatic cancer accounted for the majority of cases in exact patterns containing *KRAS*. These findings suggest that cancer-type distribution in our cohort cannot be fully explained by the presence of a germline *ATM* variant alone and may also be associated with its combination with co-occurring somatic variants.

It has been reported that *KRAS* represent a central somatic driver of pancreatic cancer [21,22,23]. The correlation between germline–somatic variant patterns and cancer-type distributions is consistent with previously reports in pancreatic cancer. In a mouse model expressing *Kras* G12D in the pancreas, partial or complete *Atm* deficiency was reported to cooperate with *Kras* G12D and increase pancreatic tumor formation, liver metastasis, and chromosomal instability [24]. This study indicates that *ATM* dysfunction and *KRAS* mutation can cooperate in the pancreas, providing biological plausibility for our finding that exact patterns containing a germline *ATM* variant and a somatic *KRAS* variant were predominantly associated with pancreatic cancer.

This study has several limitations. First, this was a cross-sectional, retrospective case–case analysis of cancer patients who underwent clinical CGP and was not designed to assess penetrance, the future risk of developing pancreatic cancer among unaffected variant carriers, or causality. Second, some exact-pattern strata included small numbers of patients, resulting in zero-cell counts and wide 95% confidence intervals. Given these small subgroup sizes and the exploratory nature of this study, formal interaction tests across germline backgrounds were not performed. Therefore, the observed differences among the germline *BRCA1*, *BRCA2*, and *ATM* backgrounds should be interpreted as descriptive findings rather than as evidence of statistical interaction or effect modification. Third, the GMT and NCC panels differed in gene coverage, which may have affected exact-pattern classification. We therefore performed C114 and panel-adjusted sensitivity analyses, in which some, but not all, major associations were maintained.

## 5. Conclusions

In patients with hereditary cancer predisposition syndromes, pancreatic cancer was associated with specific germline–somatic variant patterns of the *TP53* variant and *KRAS* variant, particularly those containing somatic *KRAS* variants. Integrated evaluation of germline and somatic variant patterns might provide additional insight into the genomic context associated with pancreatic cancer in patients with hereditary cancer predisposition syndromes.

## Figures and Tables

**Table 1:** Clinical characteristics of patients who underwent tumor–normal (T/N) paired testing.

Characteristic	Category	Germline Positive Patients	Germline Negative Patients	*p* Value
		(*n* = 853)	(*n* = 17,879)	
Age	Mean ± SD	56.5 ± 18.0	59.9 ± 16.3	<0.001
Sex	Female	457 (53.6%)	8837 (49.4%)	0.018
	Male	396 (46.4%)	9042 (50.6%)	
Panel	GMT	358 (42.0%)	7751 (43.4%)	0.426
	NCC	495 (58.0%)	10,128 (56.6%)	
Cancer type	Pancreas	200 (23.4%)	3502 (19.6%)	0.006
	Bowel	73 (8.6%)	2444 (13.7%)	<0.001
	Biliary Tract	65 (7.6%)	1524 (8.5%)	0.355
	CNS/Brain	56 (6.6%)	1281 (7.2%)	0.506
	Soft Tissue	60 (7.0%)	1233 (6.9%)	0.877
	Esophagus/Stomach	37 (4.3%)	1063 (5.9%)	0.051
	Breast	69 (8.1%)	918 (5.1%)	<0.001
	Lung	12 (1.4%)	816 (4.6%)	<0.001
	Ovary/Fallopian Tube	88 (10.3%)	724 (4.0%)	<0.001
	Head and Neck	19 (2.2%)	609 (3.4%)	0.062
	Uterus	21 (2.5%)	607 (3.4%)	0.139
	Prostate	47 (5.5%)	419 (2.3%)	<0.001
	Others	106 (12.4%)	2739 (15.3%)	0.021
Family history	Yes	689 (80.8%)	12,432 (69.5%)	<0.001
	No	126 (14.8%)	4633 (25.9%)	<0.001
	Unknown	38 (4.5%)	814 (4.6%)	0.893
Number of germline pathogenic variants	1	839 (98.4%)	0	
	2	13 (1.5%)	0	
	3	0 (0.0%)	0	
	4	1 (0.1%)	0	

Data are presented as mean ± standard deviation (SD) or number (%). *p* values represent comparisons between germline-positive and germline-negative patients. GMT, GenMineTOP Cancer Genome Profiling System; NCC, OncoGuide NCC Oncopanel System; CNS, central nervous system.

**Table 2:** Associations between germline–somatic variant patterns in pancreatic cancer.

Germline Variant	Somatic Variant	Pancreatic Cancer/Total	All Cancer/ Total	OR (95% CI)	Odds Ratio 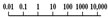	*p* Value	*q* Value
*BRCA1*	none	0/11 (0.0%)	13/120 (10.8%)	0.35 (0.02–6.21)	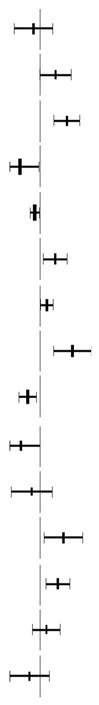	0.601	1.000
	*KRAS*	1/2 (50.0%)	12/129 (9.3%)	9.40 (0.91–97.30)		0.189	1.000
	*KRAS* & *TP53*	5/6 (83.3%)	8/125 (6.4%)	50.69 (7.32–350.95)		<0.001	0.003
	*TP53*	0/53 (0.0%)	13/78 (16.7%)	0.05 (0.00–0.78)		<0.001	0.034
*BRCA2*	none	11/57 (19.3%)	90/242 (37.2%)	0.42 (0.21–0.84)		0.010	0.276
	*BRCA2* & *KRAS*	6/7 (85.7%)	95/292 (32.5%)	8.96 (1.49–53.79)		0.007	0.207
	*KRAS*	10/18 (55.6%)	91/281 (32.4%)	2.57 (1.01–6.57)		0.044	0.974
	*KRAS* & *TP53*	23/23 (100.0%)	78/276 (28.3%)	118.85 (7.13–1980.67)		<0.001	<0.001
	*TP53*	2/31 (6.5%)	99/268 (36.9%)	0.14 (0.04–0.54)		<0.001	0.028
*ATM*	none	0/10 (0.0%)	34/71 (47.9%)	0.05 (0.00–0.92)		0.004	0.144
	*ATM*	0/2 (0.0%)	34/79 (43.0%)	0.26 (0.01–5.67)		0.507	1.000
	*ATM* & *KRAS*	8/8 (100.0%)	26/73 (35.6%)	30.47 (1.69–549.13)		<0.001	0.028
	*KRAS*	10/11 (90.9%)	24/70 (34.3%)	13.29 (2.24–78.74)		<0.001	0.028
	*KRAS* & *TP53*	2/3 (66.7%)	32/78 (41.0%)	2.38 (0.30–18.96)		0.569	1.000
	*TP53*	0/3 (0.0%)	34/78 (43.6%)	0.18 (0.01–3.69)		0.260	1.000

Associations between exact germline–somatic variant patterns and pancreatic cancer were evaluated among germline-positive patients. Within carriers of each germline variant gene, the frequency of pancreatic cancer was compared between patients with the indicated exact pattern (“with the pattern”) and those with any other exact pattern (“without the pattern”). “None” indicates no qualifying somatic variants. Events/Total indicates the number of patients with the indicated cancer type divided by the total number of patients in each group. Points indicate odds ratios (ORs), and horizontal lines indicate 95% confidence intervals (CIs). The x axis is shown on a logarithmic scale, and the vertical dashed line indicates an OR of 1. Group comparisons were performed using Fisher’s exact test or Pearson’s chi-squared test, with all tests being two-sided. *q* values were calculated using the Benjamini–Hochberg method, with *q* < 0.05 considered statistically significant after multiple-testing correction. OR, odds ratio; CI, confidence interval.

**Table 3:** Correlation between somatic variants and none/single variant in pancreatic cancer.

Germline Variant	Somatic Variant	Somatic Variant	Events /Total	Events /Total	OR (95% CI)	Odds Ratio 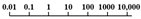	*p* Value	*q* Value
*BRCA1*	*KRAS*	None	1/2 (50.0%)	0/11 (0.0%)	23.00 (0.61–862.92)	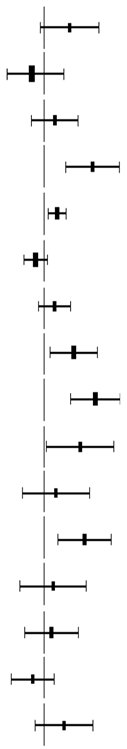	0.154	1.000
	*TP53*	None	0/53 (0.0%)	0/11 (0.0%)	0.21 (0.00–11.41)		1.000	1.000
	*KRAS* & *TP53*	*KRAS*	5/6 (83.3%)	1/2 (50.0%)	3.67 (0.20–66.31)		0.464	1.000
	*KRAS* & *TP53*	*TP53*	5/6 (83.3%)	0/53 (0.0%)	392.33 (14.20–10,839.25)		<0.001	<0.001
*BRCA2*	*KRAS*	None	10/18 (55.6%)	11/57 (19.3%)	4.99 (1.64–15.17)		0.003	0.120
	*TP53*	None	2/31 (6.5%)	11/57 (19.3%)	0.34 (0.08–1.45)		0.127	1.000
	*BRCA2* & *KRAS*	*KRAS*	6/7 (85.7%)	10/18 (55.6%)	3.51 (0.48–25.73)		0.355	1.000
	*KRAS* & *TP53*	*KRAS*	23/23 (100.0%)	10/18 (55.6%)	38.05 (2.00–722.26)		<0.001	0.024
	*KRAS* & *TP53*	*TP53*	23/23 (100.0%)	2/31 (6.5%)	554.60 (25.38–12,120.45)		<0.001	<0.001
*ATM*	*ATM* & *KRAS*	*ATM*	8/8 (100.0%)	0/2 (0.0%)	85.00 (1.32–5478.48)		0.022	0.676
	*ATM*	None	0/2 (0.0%)	0/10 (0.0%)	4.20 (0.07–267.92)		1.000	1.000
	*KRAS*	None	10/11 (90.9%)	0/10 (0.0%)	147.00 (5.35–4037.73)		<0.001	0.002
	*TP53*	None	0/3 (0.0%)	0/10 (0.0%)	3.00 (0.05–181.46)		1.000	1.000
	*ATM* & *KRAS*	*KRAS*	8/8 (100.0%)	10/11 (90.9%)	2.43 (0.09–67.58)		1.000	1.000
	*KRAS* & *TP53*	*KRAS*	2/3 (66.7%)	10/11 (90.9%)	0.24 (0.02–3.37)		0.396	1.000
	*KRAS* & *TP53*	*TP53*	2/3 (66.7%)	0/3 (0.0%)	11.67 (0.32–422.17)		0.400	1.000

Among patients carrying a germline variant in the same gene, pancreatic cancer frequencies were directly compared between nested exact pattern pairs that differed only by the addition of a single somatic variant gene. Odds ratios were calculated by comparing the pattern containing the additional somatic variant gene with the corresponding pattern without that gene. Events/Total indicates the number of patients with the indicated cancer type divided by the total number of patients in each group. Points indicate odds ratios (ORs), and horizontal lines indicate 95% confidence intervals (CIs). The x axis is shown on a logarithmic scale, and the vertical dashed line indicates an OR of 1. “None” indicates no qualifying somatic variants. Group comparisons were performed using Fisher’s exact test or Pearson’s chi-squared test, with all tests being two-sided. *q* values were calculated using the Benjamini–Hochberg method, with *q* < 0.05 considered statistically significant after multiple-testing correction. OR, odds ratio; CI, confidence interval.

## Data Availability

The datasets presented in this article are not readily available because the data are part of an ongoing study or due to limitations. Requests to access the datasets should be directed to the Center for Cancer Genomics and Advanced Therapeutics (C-CAT). The individual patient-level genomic data used in this study were derived from the C-CAT database and are not publicly available; access to these data requires an application to C-CAT (https://www.ncc.go.jp/jp/c_cat/use/flow/index.html, accessed on 17 September 2026).

## Supplementary Materials

The following supporting information can be downloaded at https://www.mdpi.com/article/10.3390/genes17091146/s1. Table S1A, panel-coverage sensitivity analysis of exact patterns; Table S1B, panel-coverage sensitivity analysis of nested patterns; Table S2A, detailed pancreatic tumor-classification labels; Table S2B, exact-pattern analysis restricted to the Pancreatic Adenocarcinoma label; Table S2C, nested-pattern analysis restricted to that label. The main supplementary results are summarized in the Supplementary Materials file.
