# Supplementary material for: The Significance of Somatic Variants Involved in the Development of Pancreatic Cancer in Patients with HBOC-Associated Germline Variants
Source: Genes (Basel). 2026 Sep 18;17(9):1146. doi: 10.3390/genes17091146 (PMC13606352; doi:10.3390/genes17091146)
Supplement: Supplementary file 1 [file genes-17-01146-s001.zip › genes-4542985-supplementary.pdf]

Supplement Table S1A. Panel-coverage sensitivity analysis for exact germline-somatic gene patterns.

Germline variant	Somatic variant	GMT with	GMT without	NCC with	NCC without	C114 OR (95% CI)	C114 *p* value	C114 *q* value (BH)	Panels	Panel-adjusted OR (95% CI)	Panel-adjusted *p* value	Panel-adjusted *q* value (BH)
BRCA1	None	0/6 (0.0%)	3/48 (6.2%)	0/6 (0.0%)	10/71 (14.1%)	0.32 (0.02-5.64)	0.608	1.000	2	0.63 (0.08-5.19)	0.601	1.000
	KRAS	1/1 (100.0%)	2/53 (3.8%)	0/1 (0.0%)	10/76 (13.2%)	9.40 (0.91-97.30)	0.189	1.000	2	8.12 (1.16-56.96)	0.178	1.000
	KRAS & *TP53*	0/2 (0.0%)	3/52 (5.8%)	5/5 (100.0%)	5/72 (6.9%)	30.15 (5.80-156.89)	<0.001	0.005	2	26.91 (5.55-130.39)	<0.001	0.006
	TP53	0/26 (0.0%)	3/28 (10.7%)	0/33 (0.0%)	10/44 (22.7%)	0.04 (0.00-0.64)	<0.001	0.013	2	0.07 (0.01-0.57)	<0.001	0.020
BRCA2	None	3/25 (12.0%)	18/73 (24.7%)	9/44 (20.5%)	71/157 (45.2%)	0.34 (0.18-0.67)	0.001	0.029	2	0.36 (0.19-0.70)	0.001	0.036
	BRCA2 & *KRAS*	1/2 (50.0%)	20/96 (20.8%)	5/5 (100.0%)	75/196 (38.3%)	8.96 (1.49-53.79)	0.007	0.149	2	9.00 (1.60-50.63)	0.007	0.168
	KRAS	3/4 (75.0%)	18/94 (19.1%)	8/15 (53.3%)	72/186 (38.7%)	2.85 (1.13-7.15)	0.022	0.426	2	2.53 (1.04-6.14)	0.046	0.899
	KRAS & *TP53*	6/6 (100.0%)	15/92 (16.3%)	20/20 (100.0%)	60/181 (33.1%)	139.34 (8.39-2315.45)	<0.001	<0.001	2	76.41 (9.14-638.42)	<0.001	<0.001
	TP53	0/13 (0.0%)	21/85 (24.7%)	2/23 (8.7%)	78/178 (43.8%)	0.12 (0.03-0.44)	<0.001	0.005	2	0.14 (0.04-0.47)	<0.001	0.003
ATM	None	0/2 (0.0%)	2/28 (7.1%)	0/9 (0.0%)	32/42 (76.2%)	0.05 (0.00-0.81)	0.002	0.048	2	0.09 (0.01-0.61)	<0.001	0.003
	ATM	0/2 (0.0%)	2/28 (7.1%)	0/1 (0.0%)	32/50 (64.0%)	0.18 (0.01-3.69)	0.260	1.000	2	0.53 (0.05-5.17)	0.407	1.000
	ATM & *KRAS*	1/1 (100.0%)	1/29 (3.4%)	7/7 (100.0%)	25/44 (56.8%)	30.47 (1.69-549.13)	<0.001	0.019	2	15.51 (1.23-194.97)	0.002	0.066
	KRAS	0/0	2/30 (6.7%)	10/11 (90.9%)	22/40 (55.0%)	13.29 (2.24-78.74)	<0.001	0.019	1	5.76 (0.93-35.45)	0.037	0.817
	KRAS & *TP53*	0/0	2/30 (6.7%)	2/3 (66.7%)	30/48 (62.5%)	2.38 (0.30-18.96)	0.569	1.000	1	1.01 (0.12-8.30)	1.000	1.000
	TP53	0/2 (0.0%)	2/28 (7.1%)	0/2 (0.0%)	32/49 (65.3%)	0.14 (0.01-2.69)	0.135	1.000	2	0.34 (0.04-2.89)	0.169	1.000

Associations between exact germline–somatic variant patterns and pancreatic cancer were evaluated among germline-positive patients. Within carriers of each germline variant gene, the proportion of pancreatic cancer was compared between patients with the indicated exact pattern (“with the pattern”) and those with all other exact patterns (“other patterns”). GMT and NCC columns show pancreatic cancer Events/Total within each panel. C114 denotes the common-114-gene analysis, in which somatic patterns in both panels were restricted to the 114 genes measured by NCC. C114 q values were calculated across all 197 observed exact-pattern comparisons using the Benjamini–Hochberg method. For the panel-adjusted analysis, when information was available from both panels, odds ratios (ORs) were estimated using the Mantel–Haenszel estimator after application of the Haldane–Anscombe correction, and 95% confidence intervals (CIs) were calculated using the Wald method on the log odds-ratio scale. Statistical significance was evaluated using the exact Cochran–Mantel–Haenszel test. When information was available from only one panel, a two-sided Fisher’s exact test or Pearson’s chi-squared test, as appropriate, was performed within that panel. Panel-adjusted q values were calculated across the 197 comparisons corresponding to Table 2. Panels indicates the number of informative panels contributing to the panel-adjusted analysis. Results based on only one informative panel were not interpreted as evidence of cross-panel robustness. “None” indicates that no somatic small-scale variants meeting the inclusion criteria were detected within the analyzed panel scope and does not indicate the absence of all genomic alterations in the tumor. OR, odds ratio; CI, confidence interval; BH, Benjamini–Hochberg; GMT, GenMineTOP Cancer Genome Profiling System; NCC, OncoGuide NCC Oncopanel System.

Supplement Table S1B. Panel-coverage sensitivity analysis for nested patterns differing by one somatic gene.

Germline variant	Somatic variant	Somatic variant	GMT with	GMT without	NCC with	NCC without	C114 OR (95% CI)	C114 *p* value	C114 *q* value (BH)	Panels	Panel-adjusted OR (95% CI)	Panel-adjusted *p* value	Panel-adjusted *q* value (BH)
BRCA1	*KRAS*	None	1/1 (100.0%)	0/6 (0.0%)	0/1 (0.0%)	0/6 (0.0%)	25.00 (0.67-934.50)	0.143	1.000	1	39.00 (0.53-2883.82)	0.143	1.000
	TP53	None	0/26 (0.0%)	0/6 (0.0%)	0/33 (0.0%)	0/6 (0.0%)	0.21 (0.00-11.10)	1.000	1.000	0	NE	NE	NE
	KRAS & *TP53*	*KRAS*	0/2 (0.0%)	1/1 (100.0%)	5/5 (100.0%)	0/1 (0.0%)	2.20 (0.15-33.14)	1.000	1.000	2	1.38 (0.18-10.71)	1.000	1.000
	KRAS & *TP53*	*TP53*	0/2 (0.0%)	0/26 (0.0%)	5/5 (100.0%)	0/33 (0.0%)	261.80 (11.11-6168.12)	<0.001	<0.001	1	737.00 (13.20-41149.57)	<0.001	<0.001
BRCA2	*KRAS*	None	3/4 (75.0%)	3/25 (12.0%)	8/15 (53.3%)	9/44 (20.5%)	6.22 (2.12-18.27)	<0.001	0.015	2	5.60 (1.96-16.02)	0.002	0.088
	TP53	None	0/13 (0.0%)	3/25 (12.0%)	2/23 (8.7%)	9/44 (20.5%)	0.33 (0.08-1.38)	0.131	1.000	2	0.38 (0.10-1.43)	0.130	1.000
	BRCA2 & *KRAS*	*KRAS*	1/2 (50.0%)	3/4 (75.0%)	5/5 (100.0%)	8/15 (53.3%)	3.20 (0.44-23.30)	0.357	1.000	2	2.54 (0.43-15.06)	0.369	1.000
	KRAS & *TP53*	*KRAS*	6/6 (100.0%)	3/4 (75.0%)	20/20 (100.0%)	8/15 (53.3%)	39.17 (2.08-737.31)	<0.001	0.015	2	19.06 (2.22-163.66)	<0.001	0.020
	KRAS & *TP53*	*TP53*	6/6 (100.0%)	0/13 (0.0%)	20/20 (100.0%)	2/23 (8.7%)	731.40 (33.67-15887.65)	<0.001	<0.001	2	352.12 (29.43-4212.49)	<0.001	<0.001
ATM	*ATM* & *KRAS*	*ATM*	1/1 (100.0%)	0/2 (0.0%)	7/7 (100.0%)	0/1 (0.0%)	119.00 (1.95-7273.73)	0.006	0.207	2	25.00 (1.13-553.01)	0.042	1.000
	ATM	None	0/2 (0.0%)	0/2 (0.0%)	0/1 (0.0%)	0/9 (0.0%)	3.29 (0.05-197.97)	1.000	1.000	0	NE	NE	NE
	KRAS	None	0/0	0/2 (0.0%)	10/11 (90.9%)	0/9 (0.0%)	161.00 (5.89-4401.07)	<0.001	0.002	1	133.00 (4.81-3674.46)	<0.001	0.008
	TP53	None	0/2 (0.0%)	0/2 (0.0%)	0/2 (0.0%)	0/9 (0.0%)	2.56 (0.04-149.45)	1.000	1.000	0	NE	NE	NE
	ATM & *KRAS*	*KRAS*	1/1 (100.0%)	0/0	7/7 (100.0%)	10/11 (90.9%)	2.43 (0.09-67.58)	1.000	1.000	1	2.14 (0.08-60.17)	1.000	1.000
	KRAS & *TP53*	*KRAS*	0/0	0/0	2/3 (66.7%)	10/11 (90.9%)	0.24 (0.02-3.37)	0.396	1.000	1	0.24 (0.02-3.37)	0.396	1.000
	KRAS & *TP53*	*TP53*	0/0	0/2 (0.0%)	2/3 (66.7%)	0/2 (0.0%)	15.00 (0.43-524.56)	0.143	1.000	1	8.33 (0.22-320.40)	0.400	1.000

Among patients carrying a germline variant in the same gene, pancreatic cancer proportions were directly compared between nested exact pattern pairs that differed only by the addition of a single somatic variant gene. Odds ratios were calculated by comparing the pattern containing the additional somatic variant gene with the corresponding pattern without that gene. GMT and NCC columns show pancreatic cancer Events/Total for the pattern with and without that additional gene within each panel. C114 denotes the common-114-gene analysis, in which somatic patterns in both panels were restricted to the 114 genes measured by NCC. C114 q values were calculated across all 205 observed nested-pattern comparisons using the Benjamini–Hochberg method. For the panel-adjusted analysis, when information was available from both panels, odds ratios (ORs) were estimated using the Mantel–Haenszel estimator after application of the Haldane–Anscombe correction, and 95% confidence intervals (CIs) were calculated using the Wald method on the log odds-ratio scale. Statistical significance was evaluated using the exact Cochran–Mantel–Haenszel test. When information was available from only one panel, a two-sided Fisher's exact test or Pearson's chi-squared test, as appropriate, was performed within that panel. Panel-adjusted q values were calculated using the Benjamini–Hochberg method with all 205 comparisons corresponding to Table 3 defined as the multiple-testing family. Of these, 88 comparisons had estimable panel-adjusted p values, whereas 117 were not estimable and were treated as $p = 1$ for multiple-testing adjustment only; their p and q values are reported as NE. Panels indicates the number of informative panels contributing to the panel-adjusted analysis. Results based on only one informative panel were not interpreted as evidence of cross-panel robustness. "None" indicates that no somatic small-scale variants meeting the inclusion criteria were detected within the analyzed panel scope and does not indicate the absence of all genomic alterations in the tumor. NE, not estimable; OR, odds ratio; CI, confidence interval; BH, Benjamini–Hochberg; GMT, GenMineTOP Cancer Genome Profiling System; NCC, OncoGuide NCC Oncopanel System.

Supplement Table S2A. Detailed tumor-classification labels among patients registered with pancreatic cancer.

Tumor-classification group	Patients	Percent of pancreatic cancer cohort	Germline positive n/200 (%)	*BRCA1*[G] n/13 (%)	*BRCA2*[G] n/101 (%)	*ATM*[G] n/34 (%)	*KRAS*[S] n/159 (%)	*TP53*[S] n/88 (%)
Pancreatic Adenocarcinoma	3024	81.7%	165/200 (82.5%)	9/13 (69.2%)	80/101 (79.2%)	33/34 (97.1%)	145/159 (91.2%)	75/88 (85.2%)
Pancreas, histology not specified	397	10.7%	9/200 (4.5%)	3/13 (23.1%)	4/101 (4.0%)	0/34 (0.0%)	7/159 (4.4%)	6/88 (6.8%)
Pancreatic neuroendocrine tumor/carcinoma	110	3.0%	10/200 (5.0%)	0/13 (0.0%)	3/101 (3.0%)	1/34 (2.9%)	4/159 (2.5%)	4/88 (4.5%)
Adenosquamous carcinoma	61	1.6%	2/200 (1.0%)	0/13 (0.0%)	2/101 (2.0%)	0/34 (0.0%)	2/159 (1.3%)	2/88 (2.3%)
Acinar cell carcinoma	51	1.4%	12/200 (6.0%)	0/13 (0.0%)	12/101 (11.9%)	0/34 (0.0%)	0/159 (0.0%)	0/88 (0.0%)
Cystic/intraductal neoplasm	32	0.9%	1/200 (0.5%)	1/13 (7.7%)	0/101 (0.0%)	0/34 (0.0%)	1/159 (0.6%)	1/88 (1.1%)
Other specified pancreatic tumor	27	0.7%	1/200 (0.5%)	0/13 (0.0%)	0/101 (0.0%)	0/34 (0.0%)	0/159 (0.0%)	0/88 (0.0%)

Detailed pancreatic tumor classifications were summarized descriptively among patients registered with pancreatic cancer. No statistical testing or q values were calculated. The distribution is shown for the overall pancreatic cancer cohort and according to the indicated germline and somatic variants. [G], germline variant; [S], somatic variant.

Supplement Table S2B. Exact-pattern sensitivity analysis restricted to the Pancreatic Adenocarcinoma database label.

Germline variant	Somatic variant	With pattern Events/Total	Other patterns Events/Total	OR (95% CI)	*p* value	*q* value (BH)
BRCA1	None	0/11 (0.0%)	9/120 (7.5%)	0.51 (0.03-9.35)	1.000	1.000
	KRAS	1/2 (50.0%)	8/129 (6.2%)	14.29 (1.34-152.55)	0.133	1.000
	KRAS & *TP53*	4/6 (66.7%)	5/125 (4.0%)	39.44 (6.74-230.73)	<0.001	0.012
	TP53	0/53 (0.0%)	9/78 (11.5%)	0.07 (0.00-1.20)	0.011	0.293
BRCA2	None	4/57 (7.0%)	76/242 (31.4%)	0.18 (0.07-0.50)	<0.001	0.011
	BRCA2 & *KRAS*	6/7 (85.7%)	74/292 (25.3%)	12.71 (2.11-76.49)	0.002	0.071
	KRAS	8/18 (44.4%)	72/281 (25.6%)	2.34 (0.91-6.01)	0.080	1.000
	KRAS & *TP53*	19/23 (82.6%)	61/276 (22.1%)	15.18 (5.24-43.98)	<0.001	<0.001
	TP53	2/31 (6.5%)	78/268 (29.1%)	0.21 (0.06-0.77)	0.005	0.151
ATM	None	0/10 (0.0%)	33/71 (46.5%)	0.05 (0.00-0.97)	0.005	0.151
	ATM	0/2 (0.0%)	33/79 (41.8%)	0.28 (0.01-5.97)	0.511	1.000
	ATM & *KRAS*	8/8 (100.0%)	25/73 (34.2%)	32.33 (1.79-583.08)	<0.001	0.021
	KRAS	10/11 (90.9%)	23/70 (32.9%)	14.15 (2.38-83.97)	<0.001	0.021
	KRAS & *TP53*	2/3 (66.7%)	31/78 (39.7%)	2.51 (0.32-19.99)	0.564	1.000
	TP53	0/3 (0.0%)	33/78 (42.3%)	0.19 (0.01-3.88)	0.267	1.000

For the tumor-classification sensitivity analysis, the pancreatic cancer outcome was restricted to cases registered under the “Pancreatic Adenocarcinoma” database label. Associations between exact germline–somatic variant patterns and pancreatic cancer were evaluated among germline-positive patients. Within carriers of each germline variant gene, the proportion of pancreatic cancer was compared between patients with the indicated exact pattern (“with the pattern”) and those with all other exact patterns (“other patterns”). “None” indicates that no somatic small-scale variants meeting the inclusion criteria were detected within the analyzed panel scope and does not indicate the absence of all genomic alterations in the tumor. Events/Total indicates the number of patients with the indicated cancer type divided by the total number of patients in each group. ORs and 95% CIs were calculated using the Wald method on the log odds-ratio scale after applying the Haldane–Anscombe correction. Group comparisons were performed using Fisher’s exact test or Pearson’s chi-squared test, with all tests being two-sided. q values were calculated using the Benjamini–Hochberg method across all 244 exact-pattern comparisons, with $q < 0.05$ considered statistically significant after multiple-testing correction. OR, odds ratio; CI, confidence interval; BH, Benjamini–Hochberg.

Supplement Table S2C. Nested-pattern sensitivity analysis restricted to the Pancreatic Adenocarcinoma database label.

Germline variant	Somatic variant	Somatic variant	Events/Total	Events/Total	OR (95% CI)	*p* value	*q* value (BH)
BRCA1	*KRAS*	None	1/2 (50.0%)	0/11 (0.0%)	23.00 (0.61-862.92)	0.154	1.000
	TP53	None	0/53 (0.0%)	0/11 (0.0%)	0.21 (0.00-11.41)	1.000	1.000
	KRAS & *TP53*	*KRAS*	4/6 (66.7%)	1/2 (50.0%)	1.80 (0.12-27.90)	1.000	1.000
	KRAS & *TP53*	*TP53*	4/6 (66.7%)	0/53 (0.0%)	192.60 (7.97-4655.43)	<0.001	0.002
BRCA2	*KRAS*	None	8/18 (44.4%)	4/57 (7.0%)	9.62 (2.57-36.04)	<0.001	0.038
	TP53	None	2/31 (6.5%)	4/57 (7.0%)	1.01 (0.20-5.04)	1.000	1.000
	BRCA2 & *KRAS*	*KRAS*	6/7 (85.7%)	8/18 (44.4%)	5.35 (0.73-39.26)	0.090	1.000
	KRAS & *TP53*	*KRAS*	19/23 (82.6%)	8/18 (44.4%)	5.35 (1.36-21.00)	0.019	0.789
	KRAS & *TP53*	*TP53*	19/23 (82.6%)	2/31 (6.5%)	51.13 (9.83-265.85)	<0.001	<0.001
ATM	*ATM* & *KRAS*	*ATM*	8/8 (100.0%)	0/2 (0.0%)	85.00 (1.32-5478.48)	0.022	0.789
	ATM	None	0/2 (0.0%)	0/10 (0.0%)	4.20 (0.07-267.92)	1.000	1.000
	KRAS	None	10/11 (90.9%)	0/10 (0.0%)	147.00 (5.35-4037.73)	<0.001	0.002
	TP53	None	0/3 (0.0%)	0/10 (0.0%)	3.00 (0.05-181.46)	1.000	1.000
	ATM & *KRAS*	*KRAS*	8/8 (100.0%)	10/11 (90.9%)	2.43 (0.09-67.58)	1.000	1.000
	KRAS & *TP53*	*KRAS*	2/3 (66.7%)	10/11 (90.9%)	0.24 (0.02-3.37)	0.396	1.000
	KRAS & *TP53*	*TP53*	2/3 (66.7%)	0/3 (0.0%)	11.67 (0.32-422.17)	0.400	1.000

For the tumor-classification sensitivity analysis, the pancreatic cancer outcome was restricted to cases registered under the “Pancreatic Adenocarcinoma” database label. Among patients carrying a germline variant in the same gene, pancreatic cancer proportions were directly compared between nested exact pattern pairs that differed only by the addition of a single somatic variant gene. Odds ratios were calculated by comparing the pattern containing the additional somatic variant gene with the corresponding pattern without that gene. Events/Total indicates the number of patients with the indicated cancer type divided by the total number of patients in each group. “None” indicates that no somatic small-scale variants meeting the inclusion criteria were detected within the analyzed panel scope and does not indicate the absence of all genomic alterations in the tumor. ORs and 95% CIs were calculated using the Wald method on the log odds-ratio scale after applying the Haldane–Anscombe correction. Group comparisons were performed using Fisher’s exact test or Pearson’s chi-squared test, with all tests being two-sided. q values were calculated using the Benjamini–Hochberg method across all 213 nested-pattern comparisons, with $q < 0.05$ considered statistically significant after multiple-testing correction. OR, odds ratio; CI, confidence interval; BH, Benjamini–Hochberg.
